# Evaluating a scalable ARCHES (Addressing Reproductive Coercion in Health Settings) model in government health facilities in Uasin Gishu county, Kenya: study protocol for a cluster-randomized controlled trial

**DOI:** 10.1186/s12978-023-01697-7

**Published:** 2023-10-17

**Authors:** Erin Pearson, Jasmine Uysal, Jamie Menzel, Chi-Chi Undie, George Odwe, Wilson Liambila, Jay G. Silverman

**Affiliations:** 1 Center On Gender Equity and Health, University of California San Diego, 9500 Gilman Drive, La Jolla, CA 92093 USA; 2 Population Council Kenya, Avenue 5, 3rd Floor, Rose Avenue, Nairobi, Kenya

**Keywords:** Global health, Contraception, Gender-based violence, Reproductive coercion, Intimate partner violence, Sexual gender-based violence, Sub-Saharan Africa, Adaptation, Kenya, Protocol

## Abstract

**Background:**

Since 2013, the World Health Organization has recommended that reproductive coercion (RC) and intimate partner violence (IPV) be addressed within reproductive health services and, in 2018, the Lancet Commission on Sexual and Reproductive Health and Rights found that RC and IPV were significant contributors to unmet need for family planning (FP) and unintended pregnancy. In Kenya, the Ministry of Health (MOH) has made reduction of unintended pregnancy and gender-based violence a primary objective. Despite this need and guidance, no clinic-based intervention models outside of the U.S. (apart from the one described here) have demonstrated efficacy to improve FP use and reduce IPV or RC thereby reducing unintended pregnancy. ARCHES (Addressing Reproductive Coercion in Health Settings) is a brief, clinic-based intervention delivered by existing FP providers aiming to: (1) Increase women’s ability to use FP without interference, (2) Provide a safe and supportive environment for IPV disclosure and referral to support services, and (3) Improve quality of FP counseling, including addressing RC and IPV. The objective of this study is to generate evidence on scaling integrated FP services (including FP, RC, and IPV) in public sector health facilities in Uasin Gishu county, Kenya via adaptation and implementation of ARCHES in partnership with the Kenya MOH.

**Methods:**

A cluster-randomized controlled trial paired with concurrent implementation science assessments will test effectiveness of the ARCHES model, adapted for scale by the Kenya MOH, in reducing unintended pregnancy. Female FP clients aged 15–49 years at selected sites will complete baseline surveys (immediately prior to receiving care), immediately post-visit exit surveys, and 6-month follow-up surveys. Provider surveys will assess changes in gender-equitable attitudes and self-efficacy to address violence reported by their clients. Costs associated with scaling ARCHES will be tracked and utilized in combination with results of the effectiveness trial to assess costs and cost-effectiveness relative to the standard of care.

**Discussion:**

This study will provide evidence of the effectiveness of a facility-based intervention to address RC and IPV within public sector FP services at scale, as adapted and implemented in Uasin Gishu county, Kenya.

*Trial registration* Trial registered on 28 September 2023 with clinicaltrials.gov NCT06059196.

**Supplementary Information:**

The online version contains supplementary material available at 10.1186/s12978-023-01697-7.

## Plain language summary

This study will document evidence of the effectiveness of the ARCHES intervention, a brief, clinic-based counselling intervention demonstrated to reduce intimate partner violence and reproductive coercion and promote women’s reproductive health, as scaled in government health facilities in Kenya. ARCHES aims to (1) decrease unintended pregnancy, (2) increase family planning uptake and use/continuation, (3) decrease experiences of reproductive coercion and intimate partner violence of women and girls aged 15–49 years seeking family planning services, and to (4) improve quality of care, (5) increase gender equitable attitudes, and (6) increase self-efficacy to provide comprehensive family planning counseling among providers trained in ARCHES.

**Supplementary Information:**

The online version contains supplementary material available at 10.1186/s12978-023-01697-7.

## Background

Approximately 37% of women in Kenya report that their most recent pregnancies were mistimed or unwanted [1]. Unintended pregnancy is a major contributor to girls leaving school, with an estimated 13,000 girls dropping out of school every year in Kenya for this reason [2]. Unintended pregnancy can lead to pregnancy termination, which is often unsafe in Kenya where abortion laws are restrictive, and is a major factor in maternal mortality, which persists at levels of 353 maternal deaths per 100,000 live births [3]. Women in Kenya also experience high rates of violence from intimate partners, with 41% of women ages 15–49 reporting ever having experienced violence from a male partner [1]. Common to many contexts, women in Kenya who report intimate partner violence (IPV) are significantly more likely than other women to report that a recent pregnancy was unintended [4]. However, unlike many other contexts, use of modern contraception in Kenya is not rare, with the 2022 Demographic and Health Survey (DHS) indicating that 57% of married women reporting modern contraceptive use [1], and women reporting IPV are no less likely to use contraception than other women [4]. This combination of indicators points to an important reality for Kenyan women: it is the success of contraception rather than its use that differs for abused women. IPV is highly associated with contraceptive failure, explaining established associations of IPV and unintended pregnancy [4].

Recent research has identified the loss of female reproductive autonomy as being a direct result of male partner behaviors that interfere with women’s attempts to use contraception via either coercion to become pregnant against her wishes or interference with her use of contraception, known as reproductive coercion (RC) [5]. Reproductive coercion has been shown to be strongly associated with unintended pregnancy among adolescent and young women independent of the effects of IPV, as well as to interact with IPV to heighten risk for unintended pregnancy beyond that seen for IPV alone [6]. Consensus regarding the critical role of RC in unintended pregnancy and poor reproductive health outcomes is mounting, with guidelines published by WHO [7] identifying reproductive coercion as a key aspect of gender-based violence (GBV) to be assessed and considered by healthcare personnel globally, particularly in family planning (FP) settings. This guidance is consistent with earlier recommendations issued by the American College of Obstetrics and Gynecology supporting assessment and addressing of both RC and IPV in contraceptive counseling in order to reduce risk for unintended pregnancy [8]. Given the high prevalence of IPV and the likely role of RC as a major driver of continued high levels of unintended pregnancy and subsequent unsafe abortion among women and girls in Kenya, there is a great need for development and testing of promising intervention models that may reduce RC and increase reproductive autonomy for women in this and other low- and middle-income countries (LMICs), particularly models that may be scalable and sustainable in such contexts.

ARCHES (Addressing Reproductive Coercion within Healthcare Settings) is a family planning clinic-based IPV/RC intervention originally developed for use in the U.S. and subsequently adapted for LMIC settings, including Kenya. ARCHES involves training existing family planning providers to (1) educate FP clients about the links between reproductive health concerns and IPV; (2) counsel clients on harm reduction behaviors to reduce risk for IPV and RC; and (3) provide clients with information on violence victimization support and related local services. Implemented within routine family planning clinic visits by existing staff, this intervention is designed to be a sustainable and scalable model to address partner violence, unintended pregnancy, and reproductive coercion among women at elevated risk for all three of these interrelated concerns. The ARCHES model has been shown in cluster randomized controlled trials in the United States and Kenya to reduce experiences of gender-based violence and improve reproductive health outcomes [9–11].

In Kenya, an initial matched-pair control trial among 659 women and girls ages 15–49 years seeking FP services from six clinics in Nairobi demonstrated that ARCHES, relative to standard-of-care FP counseling and provision, decreased the odds of leaving the clinic without a modern FP method, improved attitudes regarding RC, and increased awareness of IPV support services. Providers implemented ARCHES with high fidelity (> 80%) [11], and qualitative data from clients and providers showed high feasibility and acceptability to both parties [12, 13]. Based on this evidence and need to address high rates of GBV among FP clients, the Kenya MOH has committed to scaling ARCHES to government health facilities across Kenya via a county-by-county approach, starting in Uasin Gishu county. In this study, the research teams at the University of California, San Diego (UCSD) and Population Council Kenya are partnering with the Kenya Ministry of Health (MOH) to adapt the model to government health facilities, and then pilot, scale and evaluate the model in such health facilities in a single county, Uasin Gishu, to demonstrate effectiveness of the adapted ARCHES model prior to rolling out the model to all counties nationally.

## Methods/design

### Design overview

**Fig. 1:**
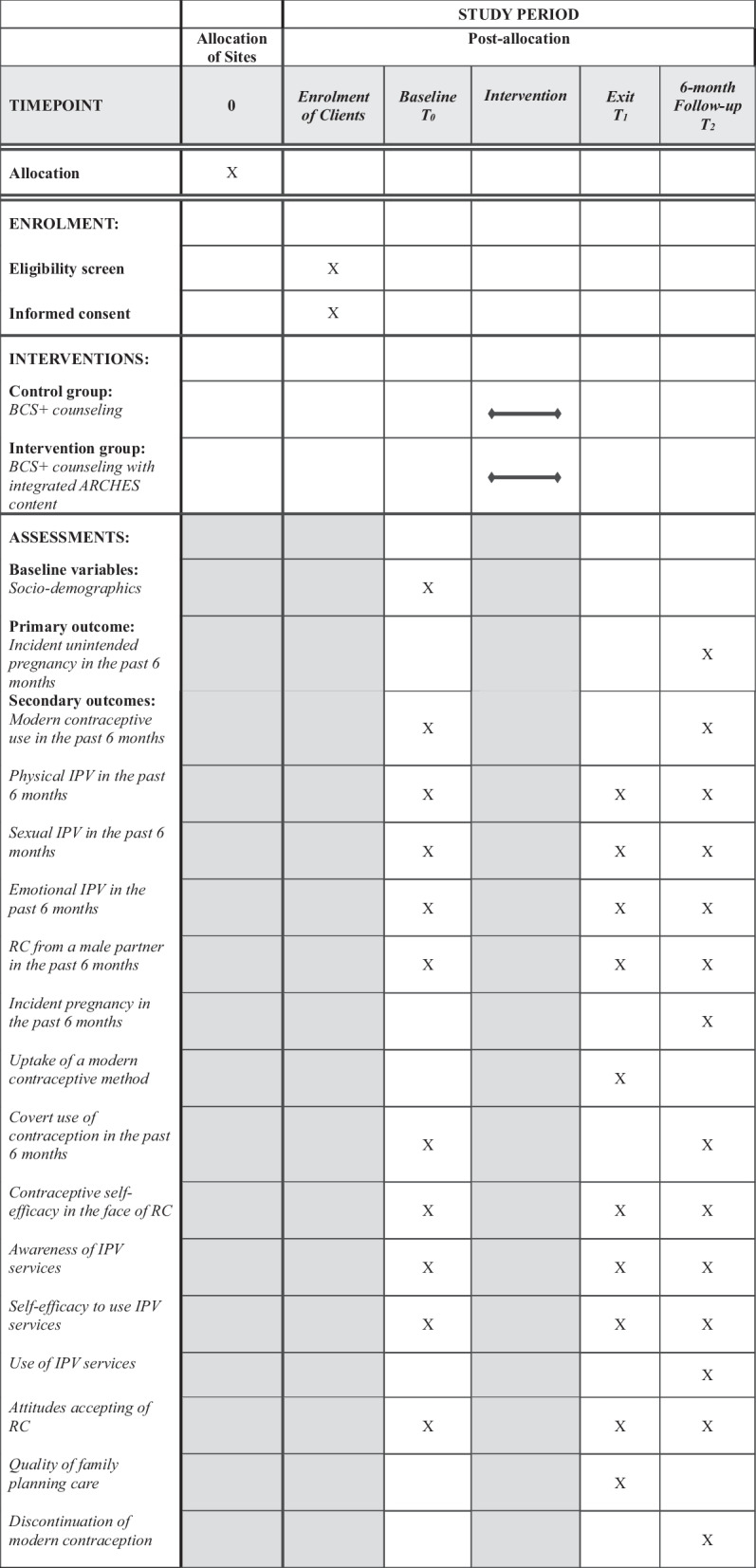
SPIRIT flow diagram

We will use a mixed methods approach to evaluate ARCHES as adapted and implemented by the Kenya MOH in government health facilities in Uasin Gishu county, Kenya. We will conduct a cluster-randomized controlled trial (cRCT) with stratified randomization based on facility type and urban/rural location to evaluate the effectiveness of the adapted ARCHES intervention on unintended pregnancy 6-months post-intervention (see Additional File 1 for items from the WHO Trial Registration Data Set). This cRCT will include 24 randomly selected health facilities (total n = 3540 female clients seeking FP care at 24 facilities; facilities are randomly assigned to either intervention or control conditions, 12 facilities per condition) in Uasin Gishu county, Kenya. Data collection from clients (women and girls aged 15–49 seeking FP services from the 24 selected health facilities) will include a baseline survey (immediately prior to receiving care at the health facility), a post-visit or exit survey (at facility immediately after receiving care), and a 6-month follow up survey (Fig. 1). A sub sample of women and girls (n = 36) who received the intervention will also be invited for 3-month follow-up interviews to understand acceptability to clients. To evaluate provider training, we will conduct: 1) pre-training, post-training, 3-month, and 6-month follow-up post-training surveys from all ARCHES trained FP providers (n ~ 60), and semi-structured interviews 3-months post-training (n = 10) and one focus group discussion (FGD) 6-months post-training (n = 1, 6–8 participants) among these providers.

### Aims

Our primary aim is to increase voluntary use of modern FP methods and ultimately decrease unintended pregnancy among female FP clients in Uasin Gishu county, Kenya by addressing barriers to successful FP uptake and use including RC, IPV, and inferior quality of healthcare (QOC) via adaptation and implementation of ARCHES.

### Setting

In May 2021, the Kenya MOH developed criteria for selection of one county (from among Kenya’s 47 counties) to participate in this research study to adapt the ARCHES model for scale. Criteria included:

- *Presence of referral facilities for GBV survivors:* Availability of supportive referral facilities was critical for implementation of ARCHES given the need to refer IPV-positive FP clients. A total of 8 counties out of 47 met the criteria and were eligible for selection.
- *High burden of GBV:* Based on GBV total survivors’ data for January–September 2020, the national mean number of GBV clients reporting to MOH facilities per county was 262 GBV clients. Counties with a reported GBV burden above the national average met this criterion. A total of 16 counties out of 47 met the criteria and were eligible for selection.
- *Low (0–30%) or medium (31–60%) modern contraceptive prevalence rate (mCPR):* Because experience of RC and IPV can impact contraceptive use, the MOH felt that ARCHES would be most impactful in counties with low or medium mCPR. A total of 34 counties out of 47 met the criteria and were eligible for selection.
- *High prevalence of teenage pregnancy:* Counties with a teenage pregnancy rate above the national average of 18% were considered because reduction of teen pregnancy is a high priority for the MOH and ARCHES is expected to reduce unintended pregnancy. A total of 21 counties out of 47 met the criteria.

Uasin Gishu was the only county in Kenya that met all four criteria, having a large GBV referral center, a high burden of GBV (542 GBV clients in 2020), a medium mCPR (56%) [14], and a high teenage pregnancy rate (22%) [14] and was, therefore, selected as the project site for adapting and scaling-up the ARCHES model in government health facilities. The government health facilities where the trial will be implemented include hospitals (100–500 FP clients/month), health centers (50–100 FP clients/month), and dispensaries (50–80 FP clients/month) that provide FP services free of cost.

### Description of intervention

#### Adapting ARCHES with the Kenya MOH

The ARCHES intervention was adapted by the Kenya MOH with technical assistance from Population Council Kenya and UCSD. The adaptation was overseen by two groups: (1) a taskforce consisting of national level MOH staff who made decisions about adaptation of the model, and (2) an implementation team consisting of Uasin Gishu county-level MOH staff who advised the taskforce regarding implementation considerations for the adaptation.

Formative research was conducted based on guidance resulting from a multi-day planning workshop led by the MOH and attended by a broad range of stakeholders that aimed to (1) identify the specific forms of reproductive coercion and other partner-specific barriers to successful contraception among women and girls seeking voluntary FP at health facilities in Uasin Gishu county, Kenya, (2) identify the physical and practice-related structures and existing provider capacities regarding counseling and referrals for clients experiencing IPV and RC (including provider attitudes and norms regarding RC and IPV), (3) guide tailoring of messaging and structure of adapted ARCHES model, and (4) assess the feasibility and current capacities to scale the intervention in health facilities across the county and nationally and specific pathways to scale considering the universal healthcare systems and decentralized Kenyan governance structures. This phase included (1) semi-structured interviews with health facility providers who offered FP services, (2) a FGD with local IPV service providers and administrators, (3) FGDs with Community Health Committees, (4) FGDs with women and girls aged 15–49 seeking FP care at government health facilities, and (5) semi-structured interviews with a subset of these same women and girls reporting past year RC or IPV. Data were analyzed and used to inform the feasibility, scalability, and acceptability of adaptation of ARCHES within existing MOH national and county-level structures. Key adaptation decisions that were made by the MOH included integrating ARCHES into the Balanced Counseling Strategy Plus (BCS +) counseling protocol, the official FP counseling approach endorsed by the MOH, and developing a mobile application that would guide providers through the counseling protocol to improve fidelity to the model. The adapted model is described in detail below.

The MOH piloted the adapted ARCHES model in five health facilities using an adaptive management approach to improve the training curriculum and refine the provider mobile application and client education materials. The pilot included assessments among providers and female FP clients to assess feasibility, acceptability, and fidelity of the adapted intervention. Data collection included: (1) pre-training and post-training surveys from all ARCHES trained FP providers, and, (2) immediately post-visit surveys (exit surveys) with women and girls aged 15–49 years receiving FP care at pilot health facilities (n = 200) to track fidelity of ARCHES implementation by trained providers, and preliminary acceptability of the model to clients. Upon completion of the pilot, the intervention materials were finalized in preparation for the trial.

#### Adapted ARCHES intervention

Health facilities assigned to the intervention group will implement ARCHES, which has been adapted by the Kenya MOH in alignment with their priorities and to ensure scalability in the public sector. As described above, the adaptation integrates ARCHES strategies in the Balanced Counseling Strategy Plus (BCS +). The BCS + is a contraceptive counseling protocol that contraceptive providers use to help clients identify suitable contraceptive methods based on their preferences and previous contraceptive experiences. The BCS + also includes systematic screening for other health services such as HIV/STI, cervical cancer, and breast cancer. The BCS + was adopted by the Kenya MOH as the standard contraceptive counseling protocol in the country, and as a result, the adaptation integrates ARCHES strategies in the BCS + counseling protocol. ARCHES strategies include training existing contraceptive providers to (1) provide education on reproductive coercion and methods/ways to use contraceptive methods discreetly if desired, (2) provide screening for reproductive coercion and intimate partner violence, (3) provide a referral to specialized services for those disclosing intimate partner violence, (4) and offer a palm-sized mini-booklet with educational information on reproductive coercion and intimate partner violence. The original ARCHES intervention relied on a three-day standalone training with existing contraceptive providers that focused solely on education about RC and IPV and building counseling skills on RC, IPV, and covert use of contraception using the GATHER counseling approach, which provided information on all available contraceptive methods, including how each method could be used covertly if the client desired. The present study will test the adapted version of ARCHES, which is implemented via integrating three hours of provider training on ARCHES-specific elements of FP counseling (as integrated within the standard BCS + counseling protocol) within the Kenya MOH’s comprehensive provider training on FP counseling and care, which includes education on reproductive anatomy, available contraceptive methods, and relevant clinical skills such as IUD and implant insertion. The training was also adapted to include instruction on use of a companion mobile application to guide providers through the counseling protocol. This six-day comprehensive training on provision of FP counseling and care includes didactic lectures, role plays, and clinical practice where trainees can practice their new counseling and clinical skills with real contraceptive clients. Providers receive post-training mentorship after returning to their health facilities to reinforce counseling and clinical skills.

#### Control condition

The control condition utilizes an active comparator, and health facilities assigned to the control group will implement standard contraceptive counseling using the BCS +. As mentioned above, the BCS + was adopted by the Kenya MOH as the standard contraceptive counseling protocol in the country, but most public sector providers have not been trained on the BCS + . As a result, the study will train existing contraceptive providers in the control group facilities on the MOH’s contraceptive training package, which will be identical to the six-day training received by intervention group providers except that content on RC and IPV will be removed and the original BCS + counseling algorithm will be used. The training will include use of a control version of the companion mobile application that guides providers through the standard BCS + counseling protocol (without ARCHES content integrated). Similar to the intervention group, control group providers will receive post-training mentorship after returning to their health facilities.

### Outcome measures

#### Primary outcome

The primary outcome is incident unintended pregnancy in the past 6 months, which is a binary variable coded to 1 if the woman reports unintended pregnancy and 0 otherwise. The study will assess the difference in self-reported unintended pregnancy in the past 6 months at the 6-month follow-up in the intervention compared to control group (single time point analysis).

#### Secondary outcomes

This study uses 15 secondary outcome measures, which can be found in Table 1 below. The IPV and RC outcomes assessments will include clients’ self-report of these forms of GBV at either baseline (at the facility prior to receiving care) or post-visit (at facility immediately after receiving care) and at six-month follow-up. We plan to use both reports at baseline and post-visit because, in the previous smaller ARCHES trial in Kenya, reporting of IPV and RC increased from baseline to post-visit among intervention clients despite no opportunity for new experiences of these forms of violence (i.e., clients had not left the clinic and partners were rarely present).

**Table 1:** Secondary outcome measures

Secondary outcome measure	Definition	Timeframe
Modern contraceptive use in the past 6 months	Change in prevalence of self-reported modern contraceptive use in the past 6 months between baseline and 6-month follow-up in intervention compared to control group (difference-in differences)	Baseline (at facility prior to receiving care) and 6-month follow-up
Physical intimate partner violence in the past 6 months	Change in prevalence of self-reported physical intimate partner violence experience in the past 6 months between combined baseline/post-visit and 6-month follow-up in intervention compared to control group (difference-in differences)	
Sexual intimate partner violence in the past 6 months	Change in prevalence of self-reported sexual intimate partner violence experience in the past 6 months between combined baseline/post-visit and 6-month follow-up in intervention compared to control group (difference-in differences)	Baseline (combined report at facility prior to receiving care and report at facility immediately after receiving care (post-visit); combined due to increased reporting post-visit) and 6-month follow-up
Emotional intimate partner violence in the past 6 months	Change in prevalence of self-reported emotional intimate partner violence experience in the past 6 months between combined baseline/post-visit and 6-month follow-up in intervention compared to control group (difference-in differences)	Baseline (combined report at facility prior to receiving care and report at facility immediately after receiving care (post-visit); combined due to increased reporting post-visit) and 6-month follow-up
Reproductive coercion from a male partner in the past 6 months	Change in prevalence of self-reported reproductive coercion experience in the past 6 months between combined baseline/post-visit and 6-month follow-up in intervention compared to control group (difference-in differences)	Baseline (combined report at facility prior to receiving care and report at facility immediately after receiving care (post-visit); combined due to increased reporting post-visit) and 6-month follow-up
Incident pregnancy in the past 6 months	Difference in self-reported pregnancy in the past 6 months at the 6-month follow-up in intervention compared to control group (single time point analysis)	6-month follow-up
Uptake of a modern contraceptive method	Difference in prevalence of self-reported modern contraceptive uptake post-visit in intervention compared to control group (single time point analysis)	Post-visit (at facility immediately after receiving care)
Covert use of contraception in the past 6 months	Change in prevalence of self-reported covert contraceptive use in the past 6 months between baseline and 6-month follow-up in intervention compared to control group (difference-in differences)	Baseline (at facility prior to receiving care) and 6-month follow-up
Contraceptive self-efficacy in the face of reproductive coercion	Change in mean self-efficacy score (range: 3–9, higher = higher self-efficacy) between baseline, post-visit, and 6-month follow-up in intervention compared to control group (difference-in differences)	Baseline (at facility prior to receiving care), Post-visit (at facility immediately after receiving care), and 6-month follow-up
Awareness of intimate partner violence services	Change in prevalence of awareness of intimate partner violence services between baseline, post-visit, and 6-month follow-up in intervention compared to control group (difference-in differences)	Baseline (at facility prior to receiving care), Post-visit (at facility immediately after receiving care), and 6-month follow-up
Self-efficacy to use intimate partner violence services	Change in mean self-efficacy score (range: 1–3, higher = higher self-efficacy) between baseline, post-visit, and 6-month follow-up in intervention compared to control group (difference-in differences)	Baseline (at facility prior to receiving care), Post-visit (at facility immediately after receiving care), and 6-month follow-up
Use of intimate partner violence services	Difference in prevalence of use of intimate partner violence services in the past 6 months at the 6-month follow-up in intervention compared to control group (single time point analysis)	6-month follow-up
Attitudes accepting of reproductive coercion	Change in mean attitude score (range: 6–12, higher = attitudes less accepting of reproductive coercion/improved attitudes) between baseline, post-visit, and 6-month follow-up in intervention compared to control group (difference-in differences)	Baseline (at facility prior to receiving care), Post-visit (at facility immediately after receiving care), and 6-month follow-up
Quality of family planning care	Difference in mean interpersonal quality of family planning scale (range: 11–55, higher = higher quality) at post-visit in intervention compared to control group (single time point analysis)	Post-visit (at facility immediately after receiving care)
Discontinuation of modern contraception	Difference in prevalence of modern contraceptive discontinuation in the past 6 months at the 6-month follow-up in intervention compared to control group (single time point analysis)	6-month follow-up

### Power and sample size

In the previous ARCHES study conducted among private sector FP clients in Nairobi, incident unintended pregnancy at six months post-intervention, the primary outcome for the present study, was 1.72% among those receiving ARCHES and 3.13% among those receiving standard-of-care FP care (AOR 0.54) (unpublished). The present study is powered to detect an AOR of 0.54 in unintended pregnancy in the intervention group compared to the control group at an alpha of 0.15, assuming intra-class correlation of 0.1 from the previous trial (unpublished) and a six-month retention rate of 80% (6-month retention rate of 82% achieved in the previous trial, unpublished). Based on these assumptions, 1770 FP clients are needed at baseline enrollment per study arm (3540 clients total, average of 148 clients per facility with 50% cluster size variation) to detect a significant difference in incident unintended pregnancy at the six-month follow-up. We assumed a binomial outcome as it is unlikely women will experience more than one unintended pregnancy within the six-month follow-up window.

Although the commonly accepted threshold is alpha = 0.05, we chose a threshold of alpha = 0.15 as acceptable for a reduction in unintended pregnancy for the present study to balance the study cost (to power the study at alpha = 0.05, the required sample size would be approximately 22,000 clients) with the Kenya MOH’s priorities, which required a focus on unintended pregnancy as the primary outcome. The MOH views 85% certainty that an observed reduction in unintended pregnancy is a true effect as reaching practical significance for guiding health policy. This approach aligns with an ever-growing consensus among statisticians that p-values should be viewed as continuous, and always considered within the context of related prior evidence, plausibility of mechanism, and stakeholder perspectives regarding risk vs. benefits of action based on the implicated change [15]. Additionally, this sample size is sufficient to show significant change among secondary outcomes at alpha = 0.05 based on results from the prior trial.

### Allocation

A total of 24 government health facilities in Uasin Gishu county were randomly selected for study participation. This cluster-randomized controlled trial used parallel assignment to two arms: (1) the intervention arm will implement the ARCHES intervention integrated in the BCS + counseling strategy, and (2) the control arm will implement standard contraceptive care using the BCS + approach. Researchers at UCSD compiled a list of the 24 facilities and stratified based on facility type (hospital, health center, or dispensary) and urban or rural location, and used SAS to generate random numbers to randomize an equal number of facilities to the intervention and control groups within these strata. All FP clients enrolled in facilities assigned to the intervention group are considered intervention group participants, and all FP clients enrolled in facilities assigned to the control group are considered control group participants.

### Participants

#### Ethics approval

This study has been approved by the University of California, San Diego Institutional Review Board (IRB) (Protocol Number: 201922S), the Population Council IRB (Protocol Number: 969), and the Kenyatta National Hospital/University of Nairobi Ethics Review Committee (Protocol Number: P369/05/2021).

#### Protocol amendments

Substantive modifications to the study protocol that impact the study procedures will be approved by the three ethics committees through formal amendments prior to implementation.

#### Recruitment

Female FP clients will be recruited from all 24 health facilities selected for the evaluation trial (n = 3540). Women and girls visiting the clinic and directed to the FP counseling station will be recruited by a research assistant (RA) hired and trained by Population Council Kenya. The RA will ask clients if they would be interested in hearing about a women’s health study being conducted at the clinic. If the client is interested, the RA will accompany the client to a separate, private room. The RA will describe the study to see if the client is interested in hearing more. If the clients expresses interest in hearing more about the study, the RA will begin eligibility screening. If eligible, the RA will administer informed consent, explaining the study, including the nature of the study, what their participation would entail, the compensation structure, and follow-up data collection. All screened potential participants will be provided with an information sheet that includes available community and health resources, including service information for local IPV services. Given the volume of FP clients seen at each selected health facility, recruitment is expected to last 2–3 months to enroll the approximately 148 FP clients required from each study site.

#### Eligibility

For female FP clients, eligibility criteria will include being: A) A health facility client seeking voluntary FP services, B) Aged 15–49 years old, C) Female, D) Able to provide informed consent, E) Able to speak and understand English, Kiswahili, or Kalenjin, F) Not sterilized (self-report), G) Not currently pregnant at baseline (self-report), H) Able to provide a safe phone number at which they can be recontacted for follow-up, and I) Not planning to move out of the area in the coming 6 months. FP providers must be A) Above age 18, B) Practicing MOH FP provider at health facility selected for intervention delivery, and C) Participate in the ARCHES FP provider training. Providers asked to participate in follow-up interviews or FGDs must have delivered ARCHES FP services to at least 25 clients.

#### Informed consent

Informed consent will be obtained in a private area in the health facility by the trained RA before any survey administration, interviews, or FGDs are conducted. As described above, participants will be notified of the purpose of the study, what is entailed in their participation, the risks and benefits of participating in the study, and clarify that participation is voluntary, consent can be withdrawn at any time during the study, there are no consequences for withdrawing from the study, and their participation is in no way tied to their ability to receive clinical services at the health facility or employment at the health facility (see Additional File 2 for Model Consent Form). Moreover, it will be reiterated at multiple points in the survey (e.g., before survey sections on any sexual behaviors or experiences of violence) that a participant is free to decline to answer any question they wish and may terminate the interview at any time. When obtaining consent for interview audio recordings, it will also be stated (and the consent form will indicate) that the recordings may be stopped at any time and that portions and/or the entire audio may be erased upon request. The RAs will be carefully trained not to apply pressure, to give the client space to express their concerns and address them, and to recognize if a participant is experiencing distress because of participating in the focus group, interview, or survey. Consent forms will be written in Kiswahili and English, and participants will provide a signature or mark to indicate their consent. In the case of women and girls who are illiterate or those who speak Kalenjin and cannot read in Kiswahili or English, we will read the consent form aloud and then question potential participants about points made in each paragraph of the consent (illiterate participants will provide a mark on the consent form rather than a signature).

We have obtained a waiver of parental consent for female FP clients aged 15–17 years old. Females aged 15–17 years (minors) are a critical population to include in this study due to their vulnerability to RC, IPV and poor reproductive health outcomes. In Kenya, minors are legally permitted to access reproductive health services without parent permission to protect their confidentiality and safety; requesting parental permission for participation in the current study (in which participation is highly connected with receipt of FP services) would be a breach of their right to confidentiality and could results in increased social and physical risk. These risks are intensified in this sensitive study about unintended pregnancy and familial/partner violence. This study also falls under the purview of the Code of Federal Regulations 45 CFR 46.408(c) and 46.116(d) which provide provisions for waiving parental permission in the case that the research providers no more than minimal risk to subjects, the waiver will not adversely affect the rights and welfare of the subjects, the research could not be practically carried out without such alteration, and that subjects will be provided with all necessary information, all of which apply to this study.

There will be no required alternative treatments or procedures for potential participants who are ineligible or decline participation in the ARCHES Kenya research. FP clients will receive the ARCHES Kenya intervention at the 12 intervention health facilities and will receive standard FP counseling services at the 12 control health facilities regardless of participation in the research per MOH protocols. Additionally, all screened potential participants will be provided with an information sheet that includes available community and health resources, including service information for IPV service providers.

### Data collection

After administering informed consent, RAs will complete the baseline survey with FP clients who agree to participate. The baseline survey will measure sociodemographic characteristics and key outcomes for difference-in-differences analysis. After completion of the baseline survey, the FP client will be walked back to the FP counseling waiting room (their previous place in line will be saved by clinic staff). When it is the participant’s turn, they will be seen for their appointment, during which the provider will deliver either the ARCHES intervention (at intervention health facilities) or standard FP counseling (at control health facilities). These services will be provided to all female FP clients at these health facilities regardless of whether they participate in the study. After the FP appointment, all participants will be directed back to the RA in a private room to complete a post-visit (exit) survey, be screened for distress, receive their compensation payment of 500 Kenyan shillings and written referrals, and schedule their six-month follow up visit. RAs will call participants to remind them of the scheduled six-month follow-up and reschedule as needed. On the day of the six-month follow-up visit, participants will return to the health facility or another location of their choosing to complete their follow-up survey. After completing their 6-month follow-up survey, participants will be given 1000 Kenyan shillings. A larger compensation payment will be given at follow-up to cover participant travel costs and to promote retention. All client surveys will be RA-administered and completed on tablets with the survey programmed in Open Data Kit (ODK), which will be programmed to require a response to all questions and other data quality controls such as including range checks for data values.

Providers trained as part of the study will complete a pre-training survey on the first day of training before any content is delivered, a post-training survey on the last day of training, and three-month and six-month follow-up surveys. Providers will not be compensated for their participation. Provider surveys will be self-administered on tablets with the survey pre-programmed in CommCare (Dimagi).

### Data management

Field supervisors will monitor RA data collection on a daily basis. Researchers at Population Council Kenya and UCSD will review electronic data for completeness and accuracy on a weekly basis. Researchers at UCSD will be responsible for variable coding for the primary analyses.

### Data analysis

Quantitative analyses to evaluate the effectiveness of ARCHES will use an intent-to-treat approach and assess the primary outcome using a multilevel mixed effects generalized linear regression model with logistic regression specifications for the binary outcome, accounting for facility-level clustering. For secondary outcomes, both single timepoint and difference-in-differences analyses will be used to compare treatment to controls, testing for an interaction between time and treatment. Difference-in-differences analyses will consider clustering within health facilities using nested random effects specifications due to repeated measurements over time of individuals nested within health facilities. Analyses will adjust for potentially relevant covariates measured at each time point. In addition to intent-to-treat analyses, we will conduct as-treated analyses to measure the effectiveness of the intervention among those who received ARCHES counseling compared to those who did not. We will also run exploratory analyses segregated by age group (age 15–24 and 25–49).

Survey items for the provider training evaluation will be tabulated and Pearson chi-square tests or T-tests (as appropriate for the outcome) at alpha < 0.05 will be used to compare responses to each item at pre-training and follow-up. For those with significant results, regression models accounting for health facility-level clustering will be used to assess the overall training effect on each outcome.

Missing client and provider data will be assessed for systematic differences between participants with missing and complete data. If systematic differences are observed, multiple imputation of missing data will be used to reduce bias.

### Dissemination

Findings will be disseminated within Kenya and internationally via presentations at scientific conferences and working group meetings, informal briefs, reports, and peer-reviewed manuscripts. Results from this evaluation will be used to inform the decision and manner of scaling the model to other counties in Kenya and other low- and middle-income countries with high rates of unintended pregnancy and GBV. Publications will be co-authored by researchers at UCSD and Population Council Kenya with authorship determined in accordance with ICMJE criteria. The de-identified client data set used for analyses will be shared on the Dyrad public data repository upon publication of findings.

### Data safety and monitoring plan

Data and safety monitoring of study participants will focus on two major safety aspects: (1) assurance that no harm comes to participants because of research participation and (2) assurance that all data collection from this study maintains the privacy of research participants. Major mechanisms of precaution include the use of confidential and anonymous data. Data from interviews and focus groups (translated transcripts) will include no personal identifiers. Survey data and audio-recordings of semi-structured interviews maintained for this study will only be labeled with unique participant identifiers; no names or other identifiers will be stored with any data collected. Population Council Kenya will maintain a list to link unique participant identifiers with personal identifiers (names, phone number) that will be stored in a secure file location separate from the database; the list will only be used for purposes of follow-up data collection, after which it will be destroyed. For qualitative focus groups and interviews, all physical data collected (i.e., audio files from focus groups and interviews) will be transcribed and translated from Kiswahili or Kalenjin to English without personal identifiers prior to electronic storage, coding, management, and analysis. Original copies of audio files that may include personal identifiers will be destroyed after transcription and translation are completed. Data management for electronic quantitative survey data collected via tablet mobile devices will include no personal identifiers and be assigned a unique study identifier. Survey data will be automatically uploaded and maintained on a secure, encrypted server. Data files will be stored on the secure online server at UCSD, which is backed up every night to minimize the likelihood of lost files. The server will be password protected and only study personnel at UCSD and Population Council will have access to the data. All research staff collecting and translating or transcribing data from this study will sign an agreement in which they agree to maintain absolute confidentiality of all participants within this research project. If any privacy or data security arrangement is violated, either through physical tampering or breach of confidentiality, the individual making the discovery will immediately notify the PIs who report the breach to the IRBs. There are some circumstances in which confidentiality may be breached by the researchers. If the participant directly informs research staff of his/her intentions of homicide or suicide, the researcher will immediately contact authorities (police or mental health) to help address the issue.

Though the study will not have a formal Data Monitoring Committee, researchers from UCSD and Population Council Kenya will monitor data to assess safety on a weekly basis. De-identified data will be analyzed by researchers at UCSD for evidence of any harms resulting from research participation. In addition, field supervisors will report any adverse events to the Population Council Kenya PI. Adverse events will be reviewed by the PIs, and adverse events resulting from research participation will be reported to the IRBs. The PIs in collaboration with the IRBs will determine whether any changes to study procedures are required or whether the trial should be terminated.

## Discussion

The ARCHES intervention has proven efficacious in increasing modern contraceptive uptake and coping with RC and IPV among private sector family planning clients in Kenya [11], but ARCHES has not previously been tested in the public sector. This study will evaluate the effectiveness of the ARCHES model at scale as adapted by the Kenya MOH for use in public sector health facilities. The ARCHES adaptation being evaluated integrates reproductive coercion and intimate partner violence content into an existing 6-day family planning provider training (compared to a three-day standalone ARCHES training) and changes the ARCHES counseling protocol from integration with the GATHER counseling protocol to integration with the BCS + counseling protocol. This is also the first adaptation of ARCHES to utilize a mobile application to guide providers through the counseling protocol, which is expected to improve fidelity as the model is scaled. The results of this study will be of interest in other LMIC settings where the ARCHES model could be implemented at scale to improve women’s reproductive autonomy.

### Supplementary Information

**Additional file 1.** Items from the WHO Trial Registration Data Set.

**Additional file 2.** Model Consent Form.

## Data Availability

Not applicable.

## Abbreviations

AOR: Adjusted odds ratio
ARCHES: Addressing Reproductive Coercion in Health Settings
BCS +: Balanced Counseling Strategy Plus
cRCT: Cluster-randomized controlled trial
DHS: Demographic and Health Survey
FGD: Focus group discussion
FP: Family planning
GATHER: Greet, Ask, Tell, Help, Explain, and Return (approach to counseling)
GBV: Gender-based violence
ICMJE: International Committee of Medical Journal Editors
IPV: Intimate partner violence
IRB: Institutional Review Board
LMIC: Low- and middle-income country
mCPR: Modern contraceptive prevalence rate
MOH: Ministry of Health
ODK: Open data kit
QOC: Quality of care
RA: Research assistant
RC: Reproductive coercion
UCSD: University of California, San Diego
