# Supplementary material for: Evaluating a scalable ARCHES (Addressing Reproductive Coercion in Health Settings) model in government health facilities in Uasin Gishu county, Kenya: study protocol for a cluster-randomized controlled trial
Source: Reprod Health. 2023 Oct 17;20:155. doi: 10.1186/s12978-023-01697-7 (PMC10583405; doi:10.1186/s12978-023-01697-7)
Supplement: Supplementary file 1 — Additional file 1. Items from the WHO Trial Registration Data Set. [file 12978_2023_1697_MOESM1_ESM.docx]

**Appendix I: Items from WHO Trial Registration Data Set**

| **Data category** | **Information** |
| --- | --- |
| Primary registry and trial identifying number | ClinicalTrials.gov NCT06059196 |
| Date of registration in primary registry | 29 September 2023 |
| Secondary identifying numbers | 201922S |
| Source(s) of monetary or material support | Bill & Melinda Gates Foundation |
| Primary sponsor | University of California, San Diego |
| Contact for public queries | Erin Pearson, PhD MPH  University of California, San Diego  Telephone: +1-254-624-6937  Email: eepearson@health.ucsd.edu |
| Contact for scientific queries | Erin Pearson, PhD MPH  University of California, San Diego |
| Public title | Adapting, Expanding, and Evaluating ARCHES (Addressing Reproductive Coercion in Health Settings) in Kenya |
| Scientific title | Adapting, Expanding, and Evaluating ARCHES (Addressing Reproductive Coercion in Health Settings) in Kenya |
| Countries of recruitment | Kenya |
| Health condition(s) or problem(s) studied | Reproductive Coercion, Intimate Partner Violence |
| Intervention(s) | Active Comparator: Control - Standard contraceptive counseling includes provider training on standard BCS+ counseling protocol and use of a companion mobile application to guide counseling  Experimental: Intervention - Integrated contraceptive counseling includes provider training on ARCHES integrated in BCS+ counseling protocol and use of a companion mobile application to guide counseling |
| Key inclusion and exclusion criteria | Inclusion Criteria:   - Seeking family planning services at a selected study facility - Aged 15-49 years old - Female - Able to provide informed consent - Able to speak and understand English, Kiswahili, or Kalenjin - Able to provide a safe phone number at which they can be recontacted for follow-up - Not planning to move out of the area in the coming 6 months   Exclusion Criteria:   - Sterilized at baseline - Pregnant at baseline |
| Study type | Interventional Allocation: randomized Intervention model: parallel assignment Masking: none  Primary purpose: treatment |
| Date of first enrolment | September 2023 |
| Target sample size | 3540 |
| Recruitment status | Recruiting |
| Primary outcome | Incident unintended pregnancy in the past 6 months  Time Frame: 6-month follow-up |
| Key secondary outcomes | Modern contraceptive use in the past 6 months  Timeframe: Baseline (at facility prior to receiving care) and 6-month follow-up |
|  | Physical intimate partner violence in the past 6 months  Timeframe: Baseline (combined report at facility prior to receiving care and report at facility immediately after receiving care (post-visit); combined due to increased reporting post-visit) and 6-month follow-up |
|  | Sexual intimate partner violence in the past 6 months  Timeframe: Baseline (combined report at facility prior to receiving care and report at facility immediately after receiving care (post-visit); combined due to increased reporting post-visit) and 6-month follow-up |
|  | Emotional intimate partner violence in the past 6 months  Timeframe: Baseline (combined report at facility prior to receiving care and report at facility immediately after receiving care (post-visit); combined due to increased reporting post-visit) and 6-month follow-up |
|  | Reproductive coercion from a male partner in the past 6 months  Timeframe: Baseline (combined report at facility prior to receiving care and report at facility immediately after receiving care (post-visit); combined due to increased reporting post-visit) and 6-month follow-up |
|  | Incident pregnancy in the past 6 months  Timeframe: 6-month follow-up |
|  | Uptake of a modern contraceptive method  Timeframe: Post-visit (at facility immediately after receiving care) |
|  | Covert use of contraception in the past 6 months  Timeframe: Baseline (at facility prior to receiving care) and 6-month follow-up |
|  | Contraceptive self-efficacy in the face of reproductive coercion  Timeframe: Baseline (at facility prior to receiving care), Post-visit (at facility immediately after receiving care), and 6-month follow-up |
|  | Awareness of intimate partner violence services  Timeframe: Baseline (at facility prior to receiving care), Post-visit (at facility immediately after receiving care), and 6-month follow-up |
|  | Self-efficacy to use intimate partner violence services  Timeframe: Baseline (at facility prior to receiving care), Post-visit (at facility immediately after receiving care), and 6-month follow-up |
|  | Use of intimate partner violence services  Timeframe: 6-month follow-up |
|  | Attitudes accepting of reproductive coercion  Timeframe: Baseline (at facility prior to receiving care), Post-visit (at facility immediately after receiving care), and 6-month follow-up |
|  | Quality of family planning care  Timeframe: Post-visit (at facility immediately after receiving care) |
|  | Discontinuation of modern contraception  Timeframe: 6-month follow-up |
