# Supplementary material for: Evaluating a scalable ARCHES (Addressing Reproductive Coercion in Health Settings) model in government health facilities in Uasin Gishu county, Kenya: study protocol for a cluster-randomized controlled trial
Source: Reprod Health. 2023 Oct 17;20:155. doi: 10.1186/s12978-023-01697-7 (PMC10583405; doi:10.1186/s12978-023-01697-7)
Supplement: Supplementary file 2 — Additional file 2. Model Consent Form. [file 12978_2023_1697_MOESM2_ESM.pdf]

Form 2.6: Informed Consent Form for baseline, exit, and 6-month follow-up surveys– Female FP Patient 15-49 Years

Title of Research Study: Adapting, Expanding, and Evaluating ARCHES (Addressing Reproductive Coercion within Healthcare Settings) within Kenya.

General Introduction

Good morning/afternoon. Thank you for taking the time to talk to me today. My name is ________________. I work with the Population Council and we are conducting a women's health study to better understand women's experiences using family planning. Many women have a difficult time using family planning or making pregnancy decisions because of their male partners. Our study is trying to make it easier for women to choose their family planning method and to have the number of children they want without any pressure from others. You will be one of 3,740 other females seeking family planning services at these health centers participating in this portion of the study. This study is being carried out in partnership with Kenya Ministry of Health and the University of California, San Diego. This study is sponsored and funded by the Bill and Melinda Gates Foundation.

You are invited to take part in a research study. Before you decide whether or not you want to participate, you need to understand why the research is being done and what it would involve. Please take the time to read along or to listen as I read the following information aloud. You may talk to others about the study if you wish. If there is anything that you find unclear, or if you are confused at any time, please stop me so I can provide more information for you. When all of your questions have been answered, and you feel that you understand this study, you will be asked if you wish to participate in the study. If you agree, we will ask you to sign the Informed Consent form which means we have provided information on the study and you understand this information. You may be given copy if you would like one. If you prefer not to have a copy at this time, please note that you can request the clinic or the study team to provide you with a copy at any time.

Purpose of the Study and Study Requirements

What is the purpose of this study? This study seeks to better understand women's experiences with their partner when using family planning or making pregnancy decisions. We also hope to help improve women's ability to choose the family planning method that works best for them and decide when to have children without pressure, violence, or fear of violence.

Why have I been invited to take part? You have been invited to take part in this study because you said you were interested in receiving family planning at your health center appointment today. As a

woman who is planning her family or using contraception and making decisions about family planning, we hope to learn from your experiences to improve access to family planning for women in Kenya

What will happen if I take part? If you agree to take part in this study, we will ask you to sign this form. You will participate in two interviews today – a longer one, and a shorter one. During the longer interview, you will also be asked to answer a series of questions on issues such as your relationship with your spouse/partner, your use of family planning, your community, and your thoughts about and experiences of violence. During the shorter interview, you will be asked questions about how your visit went with the doctor. In six months, you will be re-contacted for a follow-up survey.

How long will the interview last? The longer interview will take 30 to 40 minutes, and the shorter interview will take less than 15 minutes.

We will contact you again by phone in about 6 months from now to schedule a follow-up interview lasting about 30 to 60 minutes, if you are willing, to see how your situation has changed over time. We may also text you or call you on a monthly basis to ensure we have the correct contact information.

Confidentiality: Will my participation in the study be kept confidential?
The information that is collected during the interview will be kept private. No one will be told that you have participated in the study. The study team will protect your privacy and maintain the confidentiality of all the information that you provide. Your name or other identifiers will not be included in reports from this study. Electronic data will be stored in a password-protected computer and any paper forms will be kept in a locked cabinet dedicated to this study that only the study team can access.

Risks: What are the risks of the study? The time and effort you take to participate may be an inconvenience. You may find one or more of the questions that we ask to be upsetting or emotionally sensitive. You do not have to respond to any question that makes you uncomfortable. You may end your participation in the study, not answer a question, or leave at any time without penalty. There may be accidental loss of confidentiality, where someone not affiliated with the study may find out about your participation. Because this is a research study, there may also be some unknown risks that are currently unforeseeable. You will be informed of any significant new findings.

Benefits: What are the benefits of participating? There are no direct benefits to you for participating in the study other than a modest compensation (see additional information). You may find an indirect benefit in knowing that the information you provide will be used to improve family planning and other health services for women in Kenya.

Voluntariness: What are my rights as a research participant/participant? Your participation in this study is completely voluntary. If you decide not to participate, you will not lose any existing benefits to

which you are entitled at Ministry of Health centers. If you agree to participate in this study, you may stop at any time without penalty. If you decide to take part, you are free to refrain from answering any questions. You are free to withdraw at any time without affecting your relationship with the health center.

What are the alternatives to participating in this study?
The alternative to participation is to not participate in this study. If you chose not to participate, you will still be entitled to receive health services from the health center with no consequences.

Additional Information

What will I receive for participating? You will receive KSh 500 as a token of appreciation for your participation today and KSh 1000 for your participation in the final interview in 6 months' time. If you wish, you will be referred for additional counseling services after the interviews.

What will happen to the results of the research study? The results of the study will be discussed with our partners, including those with the authority to effect any recommended changes within the Ministry of Health. The results will also be developed into published medical and public health reports and papers for others to read and learn from.

Who has reviewed the study for ethical issues? The Institutional Review Boards of the Population Council, of the University of California, San Diego, and the Ethics Review Committee of Kenyatta National Hospital/University of Nairobi has reviewed and approved this study.

What if I need more information? If you have a concern about any aspect of the study, you should ask to speak to the researchers who will do their best to answer your questions. You may call Wilson Liambila of Population Council at Tel: +254-20-2713480; or Dr. Rose Wafula of Ministry of Health (DRMH) at Tel: +254-20-725105/6/7/8.

What if there is a problem or I have a question about my rights? Any complaint about the way you have been treated during the study or any possible harm you might suffer will be addressed. Please contact the Ministry of Health at +254 20 2717077. Complaints about any possible harm you might suffer will also be addressed. Please contact Prof. M.L. Chindia, Secretary, Kenyatta National Hospital/University of Nairobi Ethics Review Committee, P.O. Box 20723-00202 Ext 44355, Nairobi; Telephone: 020-2726300; 20 2726 3009; e-mail: uonknh_erc@uonbi.ac.ke.

Participant Statement: I have read or been read the Informed Consent for this study. I have received an explanation and understand the planned research, procedures, risks and benefits, and privacy of my

personal information. I agree to take part in this study. I understand that my participation in this study is voluntary.

Your name: ____

Your signature/thumb print: ____________________________Date:________________________

Investigator or person who conducted Informed Consent discussion: I confirm that I have personally explained the nature and extent of the planned research, study procedures, potential risks and benefits, and confidentiality of personal information.

Name of person obtaining consent: _____________________________________

Signature of person obtaining consent: ___________________Date:____________

Population Council
Avenue 5, 3rd Floor
Rose Avenue
Nairobi

University of California San Diego
Human Research Protections Program Office
TEL: 8582464777
